# Determination of profenofos in seawater and foodstuff samples after its molecularly imprinted polymer pipette-tip micro solid phase extraction optimized by response surface methodology

**DOI:** 10.1186/s13065-022-00807-z

**Published:** 2022-03-15

**Authors:** Mahsa Tamandani, Sayyed Hossein Hashemi, Massoud Kaykhaii, Ahmad Jamali Keikha, Ali Nasiriyan

**Affiliations:** 1 Department of Marine Chemistry, Faculty of Marine Science, Chabahar Maritime University, Chabahar, Iran; 2 Department of Process Engineering and Chemical Technology, Faculty of Chemistry, Gdansk University of Technology, G. Narutowicza St. 11/12, 80–233 Gdansk, Poland; 3 Department of Mechanical Engineering, Faculty of Marine Engineering, Chabahar Maritime University, Chabahar, Iran; 4 Mechanical Engineering Department, Faculty of Engineering, University of Sistan and Baluchestan, Zahedan, Iran

**Keywords:** Profenofos, Pipette tip micro-solid phase extraction, Molecularly imprinted polymer, Response surface methodology, Food samples, Seawater analysis

## Abstract

**Background:**

In this research, a molecularly imprinted polymer (MIP) was synthesized and employed as a sorbent for pipette-tip micro solid phase extraction of profenofos insecticide in seawater, rice, and fish samples. The instrument employed for quantitation was spectrophotometry.

**Results:**

Various factors affecting the microextraction protocol, including type and volume of the elution solvent, weight of MIP, pH and volume of sample solution, and number of cycles of loading and desorption were considered and optimized using one-factor-at-a-time, central composite design and Box-Behnken design. Factors optimized at: pH 4.0, amount of sorbent 2.5 mg, volume of methanol:acetic (9:1) acid as eluent 250 µL, both the number of extraction and elution cycles 5, and volume of sample 8.0 mL. At optimized conditions, an enrichment factor of 31 was achieved and the linearity range of the method was between 1.0 and 1000.0 µg/L. A good detection limit of 0.33 µg/L with a reproducibility better than 5.6% (as RSD) was observed.

**Conclusion:**

The technique showed good analytical features for determination of profenofos in seawater, rice, and fish samples. Simplicity of operation of spectrophotometry and lack of using expensive HPLC grade solvents are other points of strengths of this method. The total analysis time was about 10 min, which is far less than techniques such as HPLC. Comparison between optimization with central composite design and Box–Behnken design showed better performance of the former.

**Supplementary Information:**

The online version contains supplementary material available at 10.1186/s13065-022-00807-z.

## Introduction

Pesticides and modem agriculture have become inseparable terms. In a broad sense, pesticides are a class of chemicals to delete or combat infesting species similar insects, fungi and weeds. These chemicals are explained based on their functional class or goal organisms [1]. Organophosphate pesticides (OPs) are one type of materials explained to be esters of some acids including thiophosphoric and phosphoric acid [2]. OPs are marketed to replace recalcitrant and chlorinated pesticides [3] and they have become prevalent in USA during their first introduction to agricultural practices [4]. One of the most generally applied OP insecticides on field crops, vegetables and fruit crops is profenofos (PFF) [O-(4-bromo-2-chlorophenyl) O-ethyl S-propyl phosphoro-thioate]. It is specifically toxic for insects compared to mammals because of various metabolism of the propylthiol group [5]. PFF is considered poisonous to non-goal species consist of domestic animals, wildlife and humans despite its significant role in agriculture, [5–8]. The World Health Organization characterized PFF as Toxicity Class II (moderately poisonous), and its residues were detected in tobacco, cotton, lettuce, tomato, cucumber and beans [9, 10]. The poisonous of the pesticides on rates causes important changes in some blood biochemical factors and also in blood picture factors [11]. While, the poisonous of PFF on water vertebrates and human takes place via inhibition of the acetylcholine esterase, that observations in neuro toxicity and in addition to in instability of the erythrocyte membrane [12, 13]. For example, massive fish deaths obtained of PFF utilize observed in the US in 1998 [3]. The residue of the analyte is ingested into human using foodstuff, drinking water and is being bio-accumulated in blood, mother milk and tissue. The compound is restricted in some countries but it is still applied in other countries. So, traces of PFF should be determined by a robust and accurate analytical technique due to its general application and impacts on living organisms and media.

Several analytical protocols have been employed for the detection of pesticides in different actual samples, including gas chromatography (GC) [14], high performance liquid chromatography (HPLC) [15], capillary electrophoresis [16], and spectrophotometry [17]. There are some drawbacks using these instruments. Spectrophotometry lacks the required selectivity, while GC and HPLC are expensive techniques and capillary electrophoresis is slow for the determination of analytes.

In most cases, direct determination of traces of analytes in real samples is not easily possible due to their low concentration and interferences from their matrices [18], therefore a preconcentration/isolation step is indispensable. General extraction techniques such liquid–liquid extraction [19], solid phase extraction (SPE) [20], solid phase microextraction [21], liquid phase microextraction [22], dispersive liquid–liquid microextraction [23], molecularly imprinted polymer [24] and Fe_3_O_4_/ reduced graphene oxide nanocomposites [3] are among extraction techniques used for PFF traditionally.

Pipette-tip micro solid-phase extraction (PT-µSPE) is a simple miniaturization version of SPE, which is efficient for isolation and enrichment of target molecules in complex media. The promising method is less expensive, easier to operate and consumes a lower volume of samples and solvents than general SPE cartridges [25–27]. This technique has been used for applications such as determination of methyl dyes [26, 27] and medicines [30, 31], in seawater samples, nicotine in human plasma and cigarette [29, 31], and ciprofloxacin [30], nalidixic acid [31] and clinical and forensic toxicology samples [32].

Molecularly imprinted polymers (MIP) are synthetic polymers utilized as sorbents, which have specific recognition sites sterochemically shaped with a template molecule. They can be easily prepared by complexation between the monomers and analyte as template; therefore, the synthesized MIP can act as a selective sorbent polymer for the analyte [28, 29].

The response surface methodology (RSM) is a statistical method which is used for investigating and modeling of a signal relating to several variables. The factors affecting on protocol are called dependent variables, while the responses are dependent variables. RSM study makes an approximation relationship between input and output variables and identifies the optimum operating factors for a system in optimization or a region of the parameter field which satisfies the operating requirements. Central composite design (CCD) and Box-Behaken designs (BBD) are two main experimental designs that applied in RSM [29, 31, 33].

In this study, a novel MIP was prepared, characterized and used as a sorbent for efficient PT-µSPE of PFF from seawater, rice and fish prior to its analysis with spectrophotometry. The optimization of effective parameters on analytical signal was performed utilizing one-variable-at-a-time method, CCD and BBD. Results of CCD and BBD were compared together.

## Methods

### Apparatus

A Steroglass UV–Vis spectrophotometer model 2014 (Italy) at the wavelength of 280 nm was utilized for absorption measurements. A model 630 Metrohm (Switzerland) pH meter was applied for pH measurements. Qualitative spectra interpretations and structure investigation was performed using a Perkin-Elmer (Bucks, UK) Fourier transform infrared spectroscopy (FTIR) spectrometer. By using a scanning electron microscope (SEM), model MIRA3 TESCAN (Czech Republic), prepared MIP was investigated. Brauner-Emmett Teller (BET) surface areas, pore volume, average pore size and nitrogen adsorption/desorption analysis of MIP adsorbent was measured utilizing N_2_ physisorption with Quanta chrome Nova 2000 automated system (USA). Degassing of each sample was done in N_2_ atmosphere at 300 ^0^C for 4 h in order to obtain the BET surface areas, pore volumes and average pore sizes; sample was evacuated at –196 ^0^C. A Knauer HPLC (Germany, model: 3950) equipped with diode array detection and an EA4300F Smartline® autosampler 3950 was applied to evaluate the accuracy of the developed MIP PT-µSPE method. The analytical column was a 250 × 4.6 mm Eurospher 100–5 C_18_ with the same kind of pre-column. ChromGate V3.1.7 software was utilized for chromatographic data handing.

### Reagents

Profenofos (with 98.6% purity), methacrylic acid (MAA, 99.0%), ethylene glycol dimethacrylate (EDMA, 97%) and 2,2-azoisobutyronitrile (AIBN, 98%) were obtained from Sigma-Aldrich (St. Louis, MO, USA). All other reagents with analytical grade were received from Merck Company (Darmstadt, Germany) and utilized as received. Ultra-pure water was employed over the experiments after filtering through 0.22 mm Nylon membranes. 2000 mg/L stock solution of PFF was prepared by dissolving of suitable amount of the pesticides in 60:40 (v/v) acetonitrile/water. Daily solutions were prepared by proper dilution of the stock solution in doubly-distilled water.

### Synthesis of MIP

**Fig. 1:**
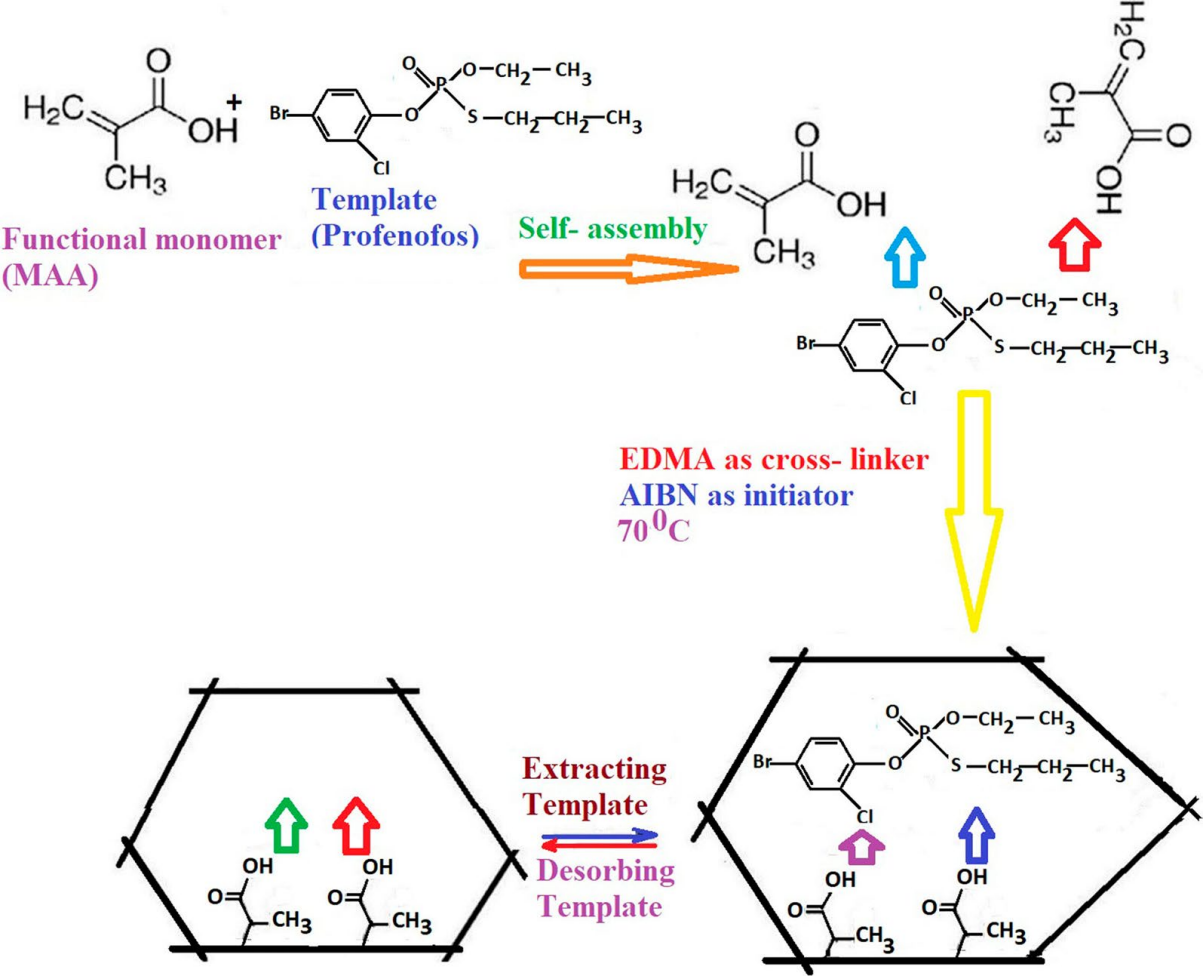
Schematic representation of synthesize procedures of MIP utilizing PFF as target analyte. PFF and MAA were poured in of acetonitrile, then added of cross-linker EDMA, and AIBN (as starter) and then polymerization was performed. Imprinting process was completed with leaching the PFF of polymer

PFF-MIP was synthesized with precipitation polymerization. At first, 1 mmol of PFF and 4 mmol of MAA were poured in 30 mL of acetonitrile. The solution was stirred for 10 min, followed by addition of 25 mmol of cross-linker EDMA, and 80 mg of AIBN (as starter). To remove oxygen, nitrogen was immerged to the regent vial for 10 min and then polymerization was performed in 60 ^0^C for 2 h. Imprinting process was completed with leaching the PFF of the above polymer utilizing methanol/ acetic acid (9:1, v/v). Figure 1 depicts the prepare of MIP. A non-imprinted polymer (NIP) was synthesized in similar conditions but in the absence of PFF to be applied as a blank.

### Extraction procedure

**Fig. 2:**
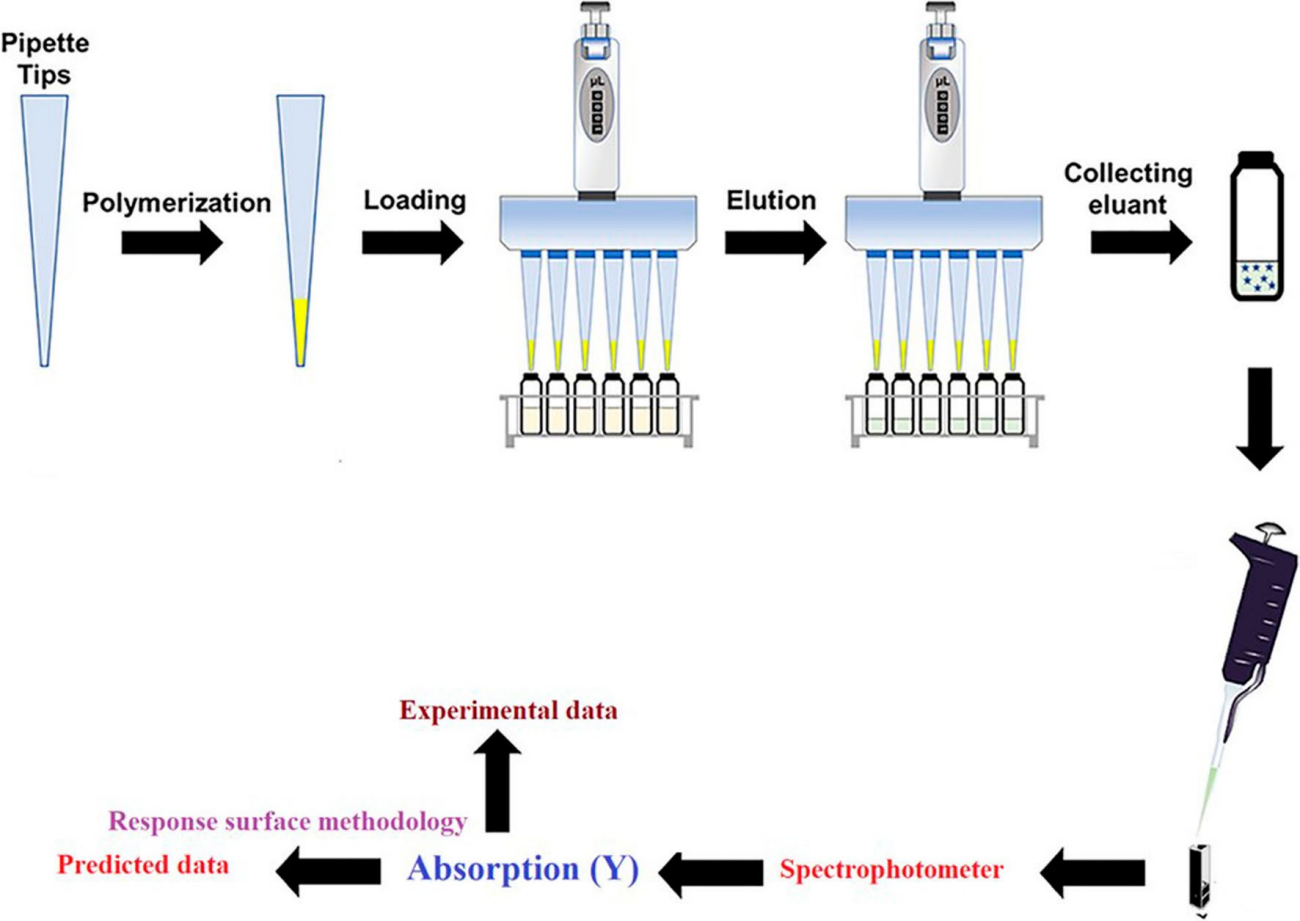
Schematic procedure of proposed MIP- PT- µSPE. MIP was added in pipette- tip and sample loaded, then, the analytes were eluted and the eluent solution was transferred to a spectrophotometer for determination

**Fig. 3:**
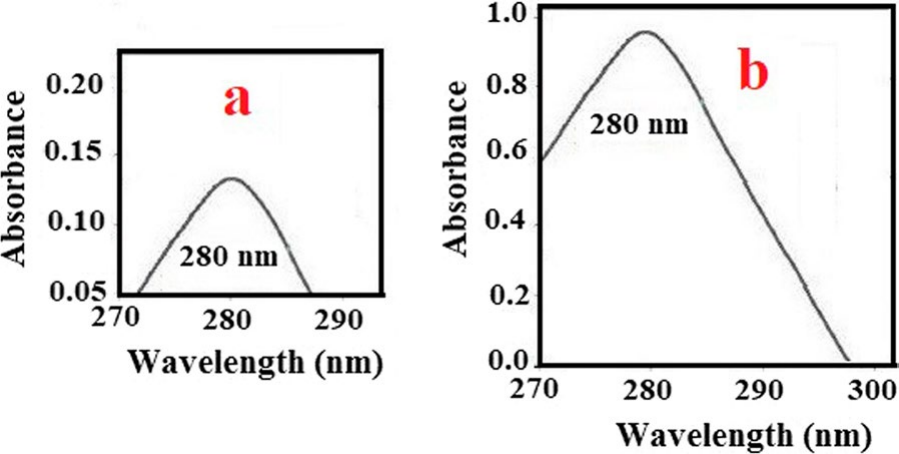
Absorbance spectra for 1000 µg/L of PFF without enrichment (**a**) and in optimum conditions after MIP extraction (**b**). The figure shown the analytical signal increased after MIP extraction

The MIP-PT-µSPE procedure is shown in Fig. 2. The first step was to pack 3.0 mg MIP in pipette- tip utilizing degreased cotton at both ends to avoid adsorbent loss. The MIP was washed by 1.0 mL of ultra-pure water applying a 10.0 mL commercial syringe. For sample loading, 1.0 mL of 100 µg/L of PFF standard with pH 4.0 was sucked into the pipette-tip for 5 times and disposed. Next, the analytes were eluted by five 250 µL portion of eluent (methanol: acetic acid (9:1)). Next, the eluent solution was transferred to a micro-cuvette of a spectrophotometer for determination. Figure 3 shows the absorbance spectra of 1000.0 µg/L of PFF before (a) and after (b) MIP-PT-µSPE. As can be seen, the analytical signal increased after MIP extraction.

### Real sample preparation

Samples of seawater were analyzed directly and without any pretreatment. Seawater samples were taken from three stations beside Chabahar Bay in southern east of Iran. Rice sample was prepared according to the procedure suggested by Bodur et al. [3] with some changes. Briefly, 0.50 g of finely powdered rice was poured in 25 mL of distilled water/methanol (1:1). After agitation, it was diluted to 100 mL with ultra-pure water and subsequently ultrasound for 20 min. After that, 10 mL of thesupernant solution was taken for analysis. To apply the proposed MIP-PT-µSPE for fish samples, Scomberomorus commerson was purchased from a local market and treated as suggested by standard EPA 3550C method [34] with a little changes. 1.0 g of powdered dried fish muscle sample was added to hexane/dichloromethane (5 mL, 1:1, v/v) and ultrasound for 25.0 min. Solution was centrifuged (15.0 min at 3500 rpm) and subsequently was decanted in conical sample tubes and preserved for the next step.

## Results and discussion

### Chromatographic conditions

Flow rate of an isocratic mobile phase (mixture of acetonitrile and water (95:5, v/v)), sample injection volume and column temperature were fixed at 1.0 mL/min, 20 µL and 20 ^0^C, respectively.

### Characterization of the MIP by SEM

**Fig. 4:**
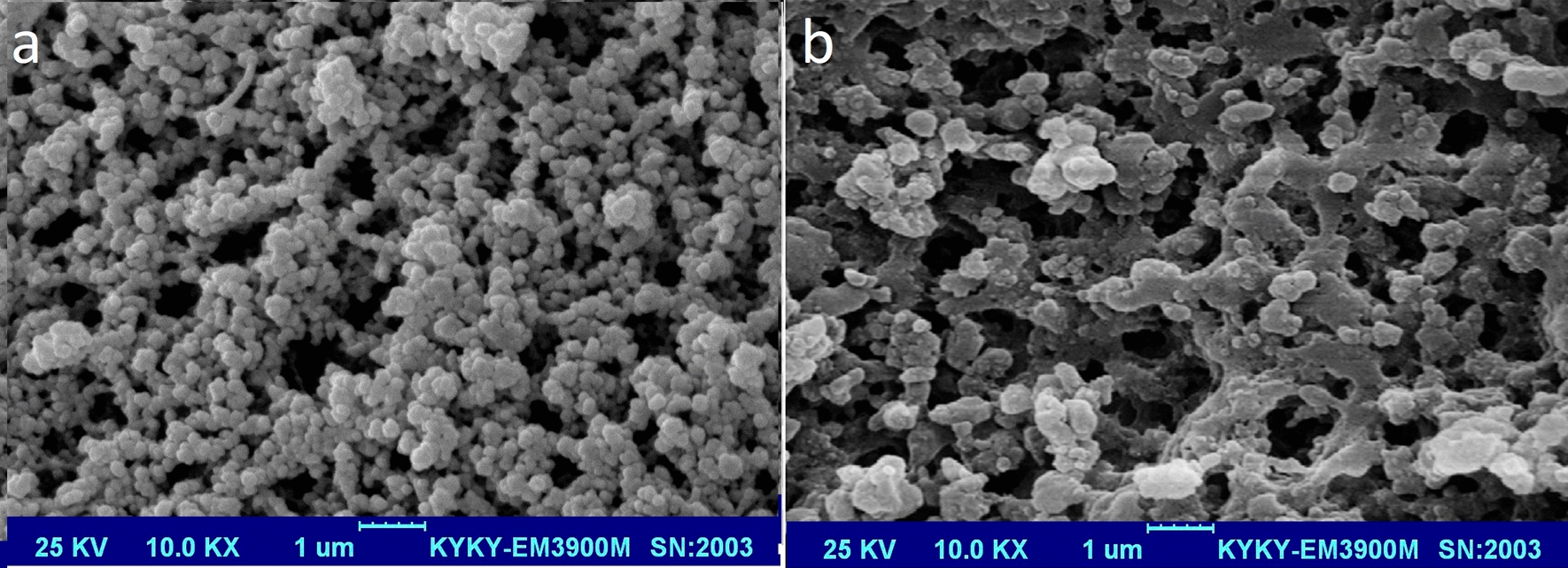
Scanning electron microscope image of the prepared MIP (**a**) and NIP (**b**) sorbent that shows which the particles were densely and uniformly synthesized and their average size is about 200 nm

The SEM images of prepared MIP and NIP are showed in Fig. 4. The creation of the pores is visible in the image, and the average size of MIP is ~ 200 nm. The SEM images revealed that the MIP seemed more denser, homogenous and uniform by more three-dimensional pores; whereas the surface of the NIP was irregular and globular without dense three-dimensional cavities.

### FTIR spectra

**Fig. 5:**
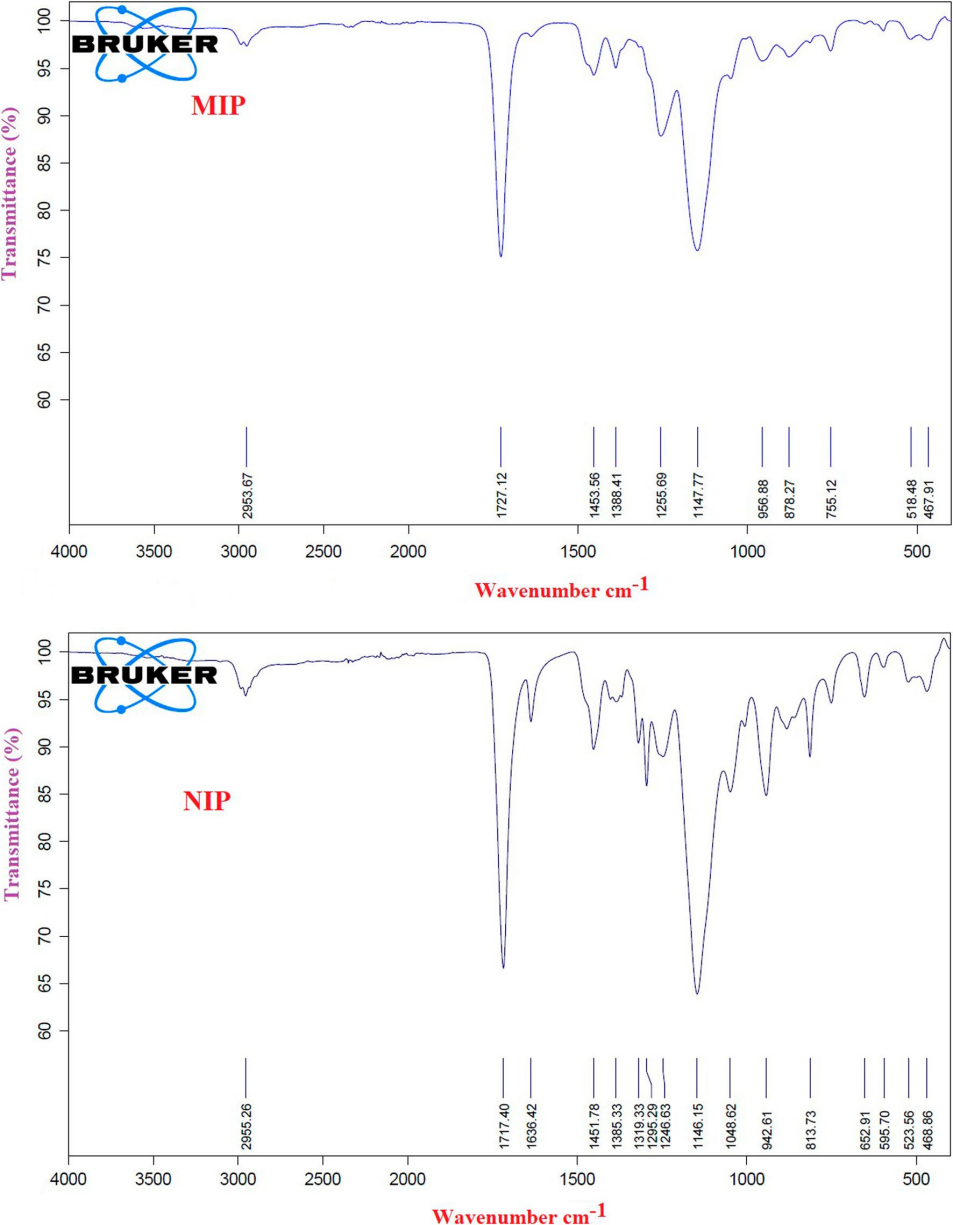
FTIR spectra of MIP and NIP

FTIR spectra were recorded for both MIP and NIP. The results of FTIR spectra of leached MIP revealed that no PFF retained on the MIP after elusion (Fig. 5). The FTIR spectra of leached MIP indicate a group of bands assigned to C-H (2954 cm^−1^), C = O (1727 cm^−1^), C-O (1255 cm^−1^) and C-N (1148 cm^−1^) that are almost similar to NIP spectra. The similarity of these spectra explains the similarity of their backbone structures. In addition it means that all PFF molecules were completely leached without any effect on the main structure of MIP.

### Nitrogen adsorption/desorption analysis

Nitrogen adsorption/desorption analysis of MIP adsorbent showed that its specific surface area is 338 m^2^/g. Average pore diameter was obtained around 0.20 µm with specific pore volume of 0.7 mL/g. The results indicate good pore structure and specific surface areas of the synthesized MIP.

### Optimization of extraction

Different parameters affecting MIP-PT-µSPE were studied to obtain optimal extraction conditions and the highest enrichment factor for the target compound by three methods of one-factor-at-a-time (OFAT), BBD and CCD. Standard solutions of 100 µg/L of PFF (by proper dilution of the stock solution in doubly-distilled water) were used for optimization, and each experiment was performed in triplicates. More significant parameters, i.e. pH, volume of eluent solvent, number of extraction cycles and number of elution cycles were optimized using CCD and BBD statistical and mathematical protocols and other parameters including volume of sample, type of elution solvent and amount of packing sorbent were optimized utilizing traditional OFAT method. Using RSM, leads to the reduction of the number of experiments and is very beneficial in terms of saving in costs and time. Full calculations, description of the parameters, equations used and statistical tables can be found as a Supplementary Data to this article. By using RSM, the number of experiments was reduced to 29 utilizing a Box-Behnken and 30 runs by using CCD. It should be noted that if all of the influencing parameters are included in RSM, equations become complicated and need plenty of time and high skill to be handled. Parameters which were optimized by OFAT are discussed in details in the following sections. For the parameters which were optimized using RSM methods, only three levels of each parameter were employed, because we were not interested in predicting extreme responses in our work. As an advantage, it was required to carry out less run trials to evaluate multiple variables and their interactions, that is more convenient and less expensive. After running, a comparison between optimization with CCD and BBD was performed and the results showed better performance of CCD. The final optimized factors selected were: pH 4.0, amount of MIP 2.5 mg, volume of eluent 250 µL, both the number of extraction and elution cycles 5, and volume of sample of 8.0 mL. The full calculation of CCD and BBD can be found in Supplementary Data.

#### Effect of the amount of packing sorbent

The mass of the packing material is a significant parameter in µSPE because of adsorption of the analyte is taking place in it. Pipette tips were filled by various masses of MIP (1.0, 1.5, 2.0 2.5 and 3.0 mg), and effect of the amount of packing material were observed. The analytical signal increased by increasing the sorbent mass up to 2.5 mg because by increasing the amount of sorbet, more sites are available, which leads to a higher signal. After 2.5 mg, the analytical signal was decreased because sample passage and elution becomes difficult. Thus, in subsequent experiments, 2.5 mg of MIP was applied as optimal amount of packing sorbent.

#### Effect of type of elution solvent

In order to elute all analyte adsorbed on the MIP by using minimum amount of the elution solvent and in a short time, several elution solvents were studied including methanol, ethanol, acetonitrile, acetic acid, methanol:acetonitrile (1:1, 2:1 and 1:2 v/v), methanol:acetic acid (1:1, 2:1, 1:2, 3:1, 6:1 and 9:1 v/v) and HCl (0.5 and 1.0 mol/L). The best efficiency was observed for methanol: acetic acid (9:1) and this solvent was selected as the suitable elution solvent for the further runs. That is probably because methanol: acetic acid (9:1) is a polar solvent with relatively high dielectric constant; so it can easily elute PFF.

#### Effect of volume of sample

The volume of sample was investigated (from 2.0 to 10.0 mL) to obtain the best analytical signal for the target compound. The absorption of the PFF increased by increasing the volume of sample from 2.0 to 8.0 mL due to reduction in spatial obstruction. For volumes over 8.0 mL, a decrease in signal observed, due to the dilution of the sample. The results explain that 8.0 mL of sample has the best response (by increasing of volume of sample more than 8.0 mL, the adsorption of PFF on the MIP is incomplete [29]) and choice as optimum sample volume.

### Validation of MIP-PT-µSPE

Under the optimal condition, the MIP-PT-µSPE technique was validated with linearity, limit of detection (LOD), limit of quantification (LOQ), intra and inter-day precision, repeatability, reproducibility and reusability. Calibration curve was obtained applying the absorbance measured at increasing spiked levels (ten levels) in the range of 1.0 to 1000.0 µg/L (with analyzing three replicates of each point). The values were subjected to regression analysis with the protocol of least squares to calculate regression coefficients of the curve. Good linearity was achieved using calibration equation A = 0.0008C + 0.1359 (where A and C are instrument’s response (analytical signal) and concentration of PFF (µg/L), respectively), with a correlation coefficient of 0.9905. Relative standard deviation (RSD) of slope and intercept of the calibration curve was 3.6 and 4.3% (n = 3), respectively. A comparison between a standard addition calibration for a fish sample spiked with 100.0 µg/L of the analyte with external calibration, showed a deviation less than 5%, that is due to the high specificity of MIP which makes it possible that only the analyte of interest is extracted. Also, the R^2^ and statistical test as the lack-of-fit fitting test was systematically utilized during full protocol validation for assessing the linearity of calibration curve and can be found in Supplementary Data. F-value and p-value of lack of fit in analysis of ANOVA were 1.89 and 0.13, respectively indicating that the data are valid. LOD based on 3S_d_ (S_d_ is the standard deviation of 10 blank measurements) was 0.33 µg/L. To calculate a best enrichment factor, the effect of the sample volume on PFF recovery was investigated. Extraction efficiency > 97% was obtained in a sample volume of 8 mL and at the volume of eluent of 250 µL. Considering recovery of 97%, by dividing the sample volume to eluent volume, an enrichment factor of 32.0 was calculated for PFF. The real enrichment factor obtained was 31.0. The intra-day precision of proposed protocol evaluated as RSD was ranged between 1.9 and 5.1% and the inter-day reproducibility was better than 4.5% in all cases. The repeatability of suggested MIP PT-µSPE, expressed as RSD was 3.1 which was studied by performing eight replicates of the spiked sample in the 50 µg/L concentration. Relate error by mean of five replicates was—4.3% for the same concentration of PFF.

A comparison of the proposed procedure with other methods for PFF determination can be found in Table 1. As can be seen, the developed method has a lower LOD, suitable recovery and RSD, and good precision. It needs less volume of eluent and the instrument used is a simple and low cost spectrophotometer. The shorter linear range in comparison with MIP coupled to GC method is due to the very high sensitivity the GC and exhaustive extraction performed. The present protocol has advantages such as simplicity, rapidity and requirement of low volume of sample for the analysis of PFF. The prepared MIP could be employed without apparent deterioration if kept in dry air for at least 12 months without reduction of its extraction ability. The repeatability for five batches of 2.5 mg of MIP was calculated to be better than 5.3%.

**Table 1:** Comparison of the suggested protocol for analysis of PFF by the published techniques

Extraction method	Instrument used	Recovery	Volume of eluent (µL)	LOD (µg/L)	Linear range (µg/L)	RSD (%)	Ref
dispersive solid phase microextraction by Fe_3_O_4_ graphene oxide nanocomposite	HPLC with ultraviolet detector and GC	96.6–103.4	80	14.2 ng/g	0.05–100 mg/kg	9.1–12.0	3
dispersive liquid–liquid microextraction	GC	88–99%	1500	0.89	3.10–1000	7	23
MIP	Cyclic voltammetry/ impedance spectroscopy	97.0–101.2	Not mentioned	5 nM	5 × 10^−8^ to 35 × 10^−4^ M	0.19	24
MIP	GC	94.0–104.0	100 mL	294	500–10,000	5.0	35
PT-MIP-µSPE	Spectrophotometry	93.9–99.7	250	0.33	1.0–1000.0	5.6	This work

#### Reusability and stability of synthesized MIP PT-µSPE

To investigate the stability and reusability of the prepared MIP, a standard solution of PFF at the concentration of 100.0 µg/L was analyzed five times with the same adsorbent. After each adsorption–desorption cycle, MIP was washed several times with methanol:acetic acid (9:1) to be sure no analyte was remained on it. There was a very slight decrease in recovery after the four adsorption-elution cycles and after five adsorption-elution process, the recovery was reduced by 5.9%. This observation proved that MIP has enough stability and reusability. The reduction in recovery was maybe because of the damage of some imprinted cavities in the adsorbent. There was no obvious change in recovery of NIP after many extraction, because no imprinted cavities exist on them.

#### Sensitivity

**Fig. 6:**
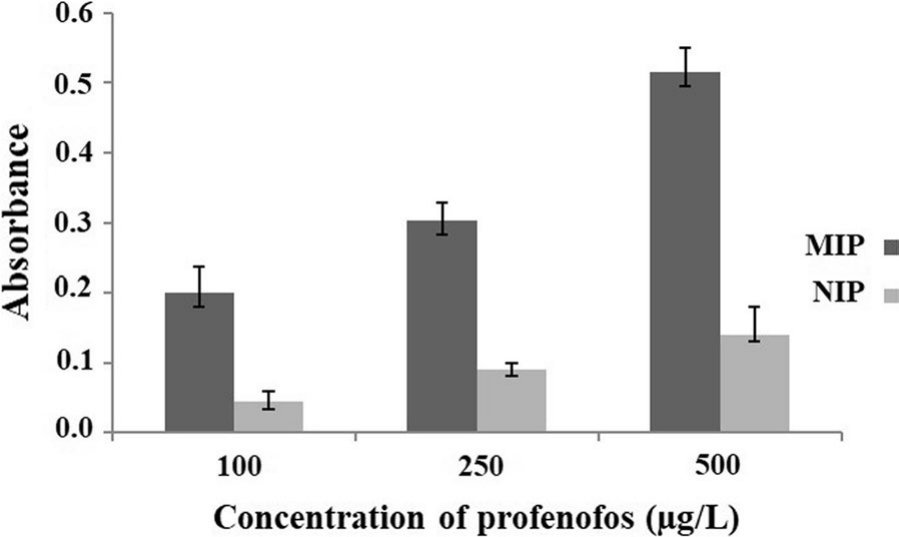
Behavior of MIP PT-µSPE and NIP PT-µSPE that proved higher sensitivity of MIP PT-µSPE

For studying the affinity of MIP against the target analyte, the absorbance of MIP and NIP PT-µSPE to PFF was investigated under the optimum conditions. The signals of MIP and NIP PT-µSPE of PFF at different concentrations showed that the MIP-PT-µSPE has a better absorbance in the range of 100 to 500 µg/L. This proves that MIP has high affinity for PFF because of the imprinting effect (Fig. 6).

#### Selectivity of MIP for PFF

The common interference that is normally present in natural sources together with PFF are chlorpyrifos (CPF), diazinon, phosalone and dichlorvos which have similar structures. To investigate the selectivity of the synthesized MIP for the extraction of PFF in samples containing these interferences, aliquots of 10 mL of 100 µg/L of PFF spiked with the same amount of the interferant was taken and analyzed with the proposed procedure. No interferences were observed for the determination of PFF, which can be related to the high selectivity of MIP toward a specific molecule.

#### Analysis of real samples

To study performance of the suggested method for real sample application, analysis of PFF in complex matrices including seawater, fish, and rice samples were performed. Moreover, these samples were spiked in three levels (10, 50 and 100 µg/L) with PFF to better investigate the matrix effect. Good recoveries were obtained in the range of 95.2 to 99.6% with RSDs better than 5.6% which indicates appropriate precision of the method. In order to be confident of the accuracy of the method, results obtained for the analysis of real samples were compared by those obtained from a standard HPLC protocol. A student’s t-test at the 95% confidence limit revealed that there is no significant difference between them in terms of accuracy. Results are summarized in Table 2.

**Table 2:** Analysis of PFF in real samples

Sample	PFF added (µg/L)	PFF found (µg/L)	Recovery (%)	RSD (%)
Seawater (sample 1, taken from Beheshti Wharf, Chabahar, Iran)	–	2.44	–	3.1
	10	12.23	97.9	2.4
	50	52.31	99.7	1.2
	100	102.18	99.7	2.6
Seawater (sample 2, taken from Kalantari Wharf, Chabahar, Iran)	–	1.53	–	2.1
	10	10.92	93.9	1.6
	50	51.09	99.1	2.7
	100	101.06	99.5	2.3
Seawater (sample 3, taken from Lipar Bay, Chabahar, Iran)	–	3.61	–	1.1
	10	12.98	93.7	1.9
	50	53.09	99.0	2.1
	100	102.7	99.1	2.9
Rice (purchased from local market)	–	0.56	–	1.1
	10	10.08	95.2	3.2
	50	50.35	99.6	4.6
	100	100.21	99.6	5.1
Fish	–	0.44	–	1.3
	10	10.39	99.5	1.7
	50	48.27	95.7	5.6
	100	97.36	96.9	4.5

## Conclusion

A new MIP was synthesized and applied as a sorbent in a PT-µSPE procedure for selective extraction of profenofos from different sample such as seawater and food samples rice and fish. A conventional spectrophotometer was used as the detection system. Low detection limit was achieved with small consumption of sample and eluent. The whole analysis time was less than 10 min. One major advantage of the suggested method is simplicity of extraction and the instrument used; still results were comparable with complicated systems such as HPLC in terms of precision, LOD and lack of interferences.

## Supplementary Information

**Additional file 1.** Additional tables.

## Data Availability

The majority of the data used to support the findings of this study are included within the article. Other data are available from the corresponding author upon request.

## Abbreviations

MP: Methylparaben
PP: Propylparaben
HPLC–UV: High performance liquid chromatography- ultraviolet
DAD: Diode array detection
UV: Ultraviolet
GC: Gas chromatography
GC–MS: Gas chromatography-mass spectrometry
SPE: Solid phase extraction
MOFs: Metal–organic frameworks
RSM: Response surface methodology
MOF PT-µSPE: Metal organic frameworks pipette tip micro-solid phase extraction
XRD: X-ray diffraction
SEM: Scanning electron microscope
ANOVA: Analysis of variance
LOD: Detection limits
EF: Enrichment factor
SPME: Solid phase microextraction
HPLC: High performance liquid chromatography
