# Supplementary material for: Determination of profenofos in seawater and foodstuff samples after its molecularly imprinted polymer pipette-tip micro solid phase extraction optimized by response surface methodology
Source: BMC Chem. 2022 Mar 15;16(1):12. doi: 10.1186/s13065-022-00807-z (PMC8922791; doi:10.1186/s13065-022-00807-z)
Supplement: Supplementary file 1 — Additional file 1. Additional tables. [file 13065_2022_807_MOESM1_ESM.docx]

**Additional data to:**

**Determination of profenofos in seawater and foodstuff samples after its molecularly imprinted polymer pipette-tip micro solid phase extraction optimized by response surface methodology**

Mahsa Tamandani^1^, Sayyed Hossein Hashemi^1^, Massoud Kaykhaii^2,*^^[[1]](#footnote-1)^, Ahmad Jamali Keikha^3^ and Ali Nasiriyan^4^

**Central composite design and Box-Behnken design**

The CCD and BBD are a type of statistical and mathematical protocols. The BBD utilizes only three levels of each parameter and at the same time a reasonable number of experimental points. In the CCD it is possible to utilize more than three levels for each factor. However, in this research, three levels were employed, because we were not interested in predicting extreme responses in our work. As a result, it was required to carry out less run trials to evaluate multiple variables and their interactions that is more convenient and less expensive. The procedures are good for experimental design, studying the effect of independent parameters on the related signal, obtaining models and investigating methods. The important variables for this extraction were pH of the solution (A or X_1_), volume of eluent solvent (B or X_2_), number of extraction cycles (C or X_3_) and number of elution cycles (D or X_4_). The pH value of the sample solution is an important variable in enrichment of PFF on the surface of MIP due to keeping of molecular state. Eluent volume is considered as other important parameter that improved the analytical signal of the analytes. The protocol of aspiration of sample solution into the conditioned pipette tip and dispensed back into the sample vial is called “an aspirating/dispensing cycle”. The number of this protocol is a critical factor for extraction in the PT-µSPE technique. In order to determine the most proper number of PFF loading cycles. The number of aspirating/dispensing cycles were investigated. Likewise, in the elution process, analytes were eluted with aspirating and dispensing. The low, middle and high levels of each factor were indicated as -1, 0, +1, respectively (Table SI1 and SI2). The design of the actual run for CCD and BBD are expressed in Table SI1 and SI2, respectively. The number of experiments required was reduced to 29 utilizing a Box-Behnken while this was 30 runs for CCD. Table SI3 explains validation of CCD and BBD models.

**Table SI1.** Central composite design of independent variables and their corresponding experimental and predicted values

| **Run** | **pH** | **Volume of Eluent Solvent (µL)** | **Extraction Cycle** | **Elution Cycle** | **Profenofos** | **Simulation** | **Error** |
| --- | --- | --- | --- | --- | --- | --- | --- |
| 1 | 5.00 | 250.00 | 5.00 | 5.00 | 0.60 | 0.61 | -2.03 |
| 2 | 4.00 | 250.00 | 3.00 | 5.00 | 0.58 | 0.58 | 0.59 |
| 3 | 5.00 | 300.00 | 3.00 | 7.00 | 0.40 | 0.40 | 0.78 |
| 4 | 3.00 | 250.00 | 5.00 | 5.00 | 0.56 | 0.56 | 0.80 |
| 5 | 4.00 | 300.00 | 5.00 | 5.00 | 0.55 | 0.55 | -0.60 |
| 6 | 4.00 | 250.00 | 7.00 | 5.00 | 0.57 | 0.58 | -1.94 |
| 7 | 3.00 | 200.00 | 3.00 | 7.00 | 0.41 | 0.40 | 3.34 |
| 8 | 4.00 | 250.00 | 5.00 | 3.00 | 0.54 | 0.54 | 0.42 |
| 9 | 3.00 | 300.00 | 7.00 | 3.00 | 0.31 | 0.31 | -0.38 |
| 10 | 3.00 | 200.00 | 3.00 | 3.00 | 0.34 | 0.35 | -2.30 |
| 11 | 5.00 | 300.00 | 7.00 | 7.00 | 0.42 | 0.42 | -0.02 |
| 12 | 5.00 | 200.00 | 7.00 | 3.00 | 0.40 | 0.40 | 0.26 |
| 13 | 4.00 | 250.00 | 5.00 | 5.00 | 0.63 | 0.64 | -1.53 |
| 14 | 3.00 | 200.00 | 7.00 | 7.00 | 0.35 | 0.36 | -4.15 |
| 15 | 5.00 | 300.00 | 7.00 | 3.00 | 0.38 | 0.38 | -1.08 |
| 16 | 3.00 | 300.00 | 3.00 | 7.00 | 0.34 | 0.35 | -2.64 |
| 17 | 3.00 | 300.00 | 7.00 | 7.00 | 0.35 | 0.34 | 2.95 |
| 18 | 5.00 | 200.00 | 3.00 | 7.00 | 0.43 | 0.44 | -1.56 |
| 19 | 4.00 | 250.00 | 5.00 | 5.00 | 0.64 | 0.64 | 0.06 |
| 20 | 4.00 | 250.00 | 5.00 | 5.00 | 0.66 | 0.64 | 3.09 |
| 21 | 5.00 | 200.00 | 3.00 | 3.00 | 0.38 | 0.38 | -0.20 |
| 22 | 5.00 | 300.00 | 3.00 | 3.00 | 0.35 | 0.34 | 1.88 |
| 23 | 4.00 | 250.00 | 5.00 | 5.00 | 0.65 | 0.64 | 1.60 |
| 24 | 4.00 | 250.00 | 5.00 | 7.00 | 0.57 | 0.58 | -1.74 |
| 25 | 4.00 | 200.00 | 5.00 | 5.00 | 0.58 | 0.58 | -0.75 |
| 26 | 3.00 | 300.00 | 3.00 | 3.00 | 0.30 | 0.30 | -1.00 |
| 27 | 4.00 | 250.00 | 5.00 | 5.00 | 0.66 | 0.64 | 3.09 |
| 28 | 3.00 | 200.00 | 7.00 | 3.00 | 0.34 | 0.33 | 1.90 |
| 29 | 4.00 | 250.00 | 5.00 | 5.00 | 0.62 | 0.64 | -3.16 |
| 30 | 5.00 | 200.00 | 7.00 | 7.00 | 0.45 | 0.44 | 2.79 |

**Table SI2.** Box-Behnken design of independent variables and their corresponding experimental and predicted values

| **Run** | **pH** | **Volume of Eluent Solvent (µL)** | **Extraction Cycle** | **Elution Cycle** | **Profenofos** | **Simulation** | **Error** |
| --- | --- | --- | --- | --- | --- | --- | --- |
| 1 | 5.00 | 300.00 | 5.00 | 5.00 | 0.54 | 0.53 | 2.16 |
| 2 | 5.00 | 250.00 | 5.00 | 3.00 | 0.55 | 0.54 | 2.27 |
| 3 | 5.00 | 250.00 | 5.00 | 7.00 | 0.59 | 0.59 | 0.14 |
| 4 | 4.00 | 250.00 | 5.00 | 5.00 | 0.65 | 0.64 | 1.23 |
| 5 | 4.00 | 200.00 | 3.00 | 5.00 | 0.52 | 0.51 | 1.12 |
| 6 | 4.00 | 200.00 | 5.00 | 7.00 | 0.54 | 0.53 | 2.62 |
| 7 | 4.00 | 250.00 | 7.00 | 7.00 | 0.56 | 0.56 | -0.30 |
| 8 | 4.00 | 300.00 | 7.00 | 5.00 | 0.50 | 0.51 | -2.17 |
| 9 | 4.00 | 250.00 | 3.00 | 3.00 | 0.52 | 0.52 | 0.32 |
| 10 | 4.00 | 250.00 | 5.00 | 5.00 | 0.64 | 0.64 | -0.31 |
| 11 | 4.00 | 250.00 | 5.00 | 5.00 | 0.63 | 0.64 | -1.91 |
| 12 | 4.00 | 250.00 | 7.00 | 3.00 | 0.53 | 0.53 | 0.00 |
| 13 | 5.00 | 250.00 | 7.00 | 5.00 | 0.57 | 0.57 | -0.73 |
| 14 | 4.00 | 300.00 | 5.00 | 7.00 | 0.48 | 0.48 | 0.87 |
| 15 | 5.00 | 200.00 | 5.00 | 5.00 | 0.49 | 0.51 | -3.74 |
| 16 | 4.00 | 200.00 | 5.00 | 3.00 | 0.46 | 0.46 | 0.18 |
| 17 | 4.00 | 300.00 | 3.00 | 5.00 | 0.45 | 0.46 | -3.15 |
| 18 | 3.00 | 250.00 | 5.00 | 7.00 | 0.48 | 0.50 | -3.65 |
| 19 | 3.00 | 200.00 | 5.00 | 5.00 | 0.46 | 0.47 | -2.54 |
| 20 | 3.00 | 300.00 | 5.00 | 5.00 | 0.44 | 0.42 | 4.16 |
| 21 | 4.00 | 250.00 | 5.00 | 5.00 | 0.67 | 0.64 | 4.18 |
| 22 | 4.00 | 250.00 | 3.00 | 7.00 | 0.55 | 0.55 | 0.00 |
| 23 | 3.00 | 250.00 | 3.00 | 5.00 | 0.50 | 0.49 | 1.83 |
| 24 | 3.00 | 250.00 | 5.00 | 3.00 | 0.48 | 0.49 | -1.22 |
| 25 | 5.00 | 250.00 | 3.00 | 5.00 | 0.57 | 0.57 | -0.44 |
| 26 | 4.00 | 250.00 | 5.00 | 5.00 | 0.62 | 0.64 | -3.55 |
| 27 | 4.00 | 300.00 | 5.00 | 3.00 | 0.47 | 0.48 | -1.95 |
| 28 | 4.00 | 200.00 | 7.00 | 5.00 | 0.50 | 0.49 | 1.83 |
| 29 | 3.00 | 250.00 | 7.00 | 5.00 | 0.52 | 0.51 | 1.44 |

**Table SI3.** Validation of the model

| **pH** | **Volume of eluent solvent (µL)** | **Extraction cycle** | **Elution cycle** | **Experimental absorbance** | **CCD absorbance** | **CCD error** | **BB absorbance** | **BB error** |
| --- | --- | --- | --- | --- | --- | --- | --- | --- |
| 4.5 | 250 | 5 | 6 | 0.60 | 0.63 | 5.00 | 0.64 | 6.67 |
| 3.5 | 275 | 3 | 5 | 0.47 | 0.52 | 10.64 | 0.51 | 8.51 |
| 4.5 | 275 | 7 | 5 | 0.62 | 0.56 | 9.68 | 0.59 | 4.84 |
| 4 | 250 | 5 | 8 | 0.55 | 0.49 | 10.91 | 0.54 | 1.82 |
| 4.5 | 250 | 8 | 4 | 0.46 | 0.48 | 4.35 | 0.52 | 13.04 |
| 4.5 | 325 | 5 | 5 | 0.44 | 0.46 | 4.55 | 0.42 | 4.55 |
| 4 | 175 | 5 | 5 | 0.48 | 0.50 | 4.17 | 0.43 | 10.42 |
| 5.5 | 250 | 6 | 6 | 0.52 | 0.54 | 3.85 | 0.55 | 5.77 |
| 4 | 200 | 8 | 5 | 0.41 | 0.44 | 7.32 | 0.43 | 4.88 |

In a system including four significant independent variables, predicted absorption using the quadratic equation (as second degree polynomial equation) for CCD and BBD can be calculated as indicated in eq. SI1.

Y= β_0_ + ∑ β_i_ X_i_ + ∑ β_ii_ X_ii_ + ∑ β_ij_ X_i_ X_j_ + ε (eq. SI1)

In eq. 1, Y is predicted absorption (process response or output or dependent variable), i and j are the index numbers for pattern, β_0_ is X_1_, X_2_, X_3_ and X_4_ are the coded independent variables, β_i_ is the linear effect, β_ii_ is the quadratic effect, β_ij_ proves the coefficient of the interaction factor, ε is the random error or allows for description or uncertainties between predicated and detected values [1,2].

A multiple regression analysis is carried out to obtain the coefficients and the equation can be applied to predict the absorbance.

Y (for CCD) = - 2.82891 + (0.44027 × A) + (0.013447 × B) + (0.12824 × C) + (0.21581 × D) + (3.75000 × 10^-5^ × A × B) + (4.06250 × 10^-3^ × A × C) + (9.37500 × 10^-4^ × A × D) + (5.62500 × 10^-5^ × B × C) – (6.25000 × 10^-6^ × B × D) – (1.09375 × 10^-3^ × C × D) - (0.055789 × A^2^) – (2.83158 × 10^-5^ × B^2^) – (0.015197 × C^2^) – (0.020197 × D^2^) (eq. SI2)

Y (for BBD) = - 3.21650 + (0.40383 × A) + (0.018650 × B) + (0.084167 × C) + (0.17167 × D) + (3.50000 × 10^-4^ × A × B) – (2.50000 × 10^-3^ × A × C) + (5.00000× 10^-3^ × A × D) + (1.75000 × 10^-4^ × B × C) - )1.75000 × 10^-4^ × B × D( + (0.000000 × C × D) – (0.058500 × A^2^) – (4.04000 × 10^-5^ × B^2^) – (0.011500 × C^2^) – (0.014000 × D^2^) (eq. SI3)

By solving these equation systems for the condition of ∂ (Y)/ ∂ (A) =0, ∂ (Y)/ ∂ (B) =0, ∂ (Y)/ ∂ (C) =0, ∂ (Y)/ ∂ (D) =0, the critical point in the CCD and BBD are calculated. The way of achieving these critical points has been explained applying Santelli et al. [3, 4]. The summary of the analytes of variance (ANOVA) are excessed in Table SI4 and SI5 (for BBD and CCD). The obtained data for the critical point are explains: pH (A) = 4.26 (for CCD) and 4.32 (for BBD), volume of eluent (B) = 244.74 (for CCD) and 248.95 µL (for BBD), the number of extraction cycles (C) = 5.05 (for CCD) and 5.09 (for BBD) and the number of elution cycles (D) = 5.27 (for CCD) and 5.35 (BBD) for PFF.

Reasons for optimal amount of parameters can be explained as: The reduction of absorbance in lower or higher pH could be attributed to hydrolysis in strongly acidic or alkaline solutions. When the volume of eluent is greater than optimal volume, a reduction in absorbance observed because of the decrease in mass transfer in an effective way. In a low volume of eluent, the extraction of PFF cannot be obtained efficiently and in larger volumes, the concentration of analytes can be decreased due to inconsequence of dilution and enrichment. Also, in lower number of extraction and elution, the adsorption and desorption of PFF on the MIP is incomplete, respectively and in higher their number, the analyte can back to solution and sorbent.

**Table SI4.** ANOVA analysis for response profenofos indicating sum of squares, mean square and percent contribution (CCD design)

| **Source** | **Sum of squares** | **df** | **Mean square** | **F- Value** | **p-value Prob > F** | **% PC= (SS/ ∑ SS) × 100** |
| --- | --- | --- | --- | --- | --- | --- |
| Model | 0.44 | 14 | 0.031 | 166.66 | < 0.0001 |  |
| A-pH | 0.014 | 1 | 0.014 | 76.71 | < 0.0001 | 18.42 |
| B-Volume of Eluent Solvent | 0.0044 | 1 | 0.0044 | 23.12 | 0.0002 | 5.79 |
| C-Extraction Cycle | 8.89 × 10^-5^ | 1 | 8.89 × 10^-5^ | 0.4719 | 0.50 | 0.12 |
| D-Elution Cycle | 0.0080 | 1 | 0.0080 | 42.58 | < 0.0001 | 10.53 |
| AB | 5.62 × 10^-5^ | 1 | 5.62 × 10^-5^ | 0.30 | 0.59 | 0.074 |
| AC | 0.0011 | 1 | 0.0011 | 5.61 | 0.03 | 1.45 |
| AD | 5.62 × 10^-5^ | 1 | 5.62 × 10^-5^ | 0.30 | 0.59 | 0.074 |
| BC | 0.00051 | 1 | 0.00051 | 2.69 | 0.12 | 0.67 |
| BD | 6.25 × 10^-6^ | 1 | 6.25 × 10^-6^ | 0.033 | 0.86 | 0.0082 |
| CD | 0.00031 | 1 | 0.00031 | 1.63 | 0.22 | 0.41 |
| A^2 | 0.0082 | 1 | 0.0081 | 42.81 | < 0.0001 | 10.79 |
| B^2 | 0.013 | 1 | 0.013 | 68.92 | < 0.0001 | 17.10 |
| C^2 | 0.0096 | 1 | 0.0096 | 50.82 | < 0.0001 | 12.63 |
| D^2 | 0.017 | 1 | 0.017 | 89.77 | < 0.0001 | 22.37 |
| Residual | 0.0028 | 15 | 0.00019 |  |  |  |
| Lack of Fit | 0.0015 | 10 | 0.00015 | 0.56 | 0.80 |  |
| Pure Error | 0.0013 | 5 | 0.00027 |  |  |  |

% PC= Percent contribution, SS: sum of squares

**Table SI5.** ANOVA analysis for response profenofos indicating sum of squares, mean square and percent contribution (Box-Behnken design)

| **Source** | **Sum of squares** | **df** | **Mean square** | **F-value** | **p-value Prob > F** | **% PC= (SS/ ∑ SS) × 100** |
| --- | --- | --- | --- | --- | --- | --- |
| Model | 0.11 | 14 | 0.0078 | 28.37 | < 0.0001 |  |
| A-pH | 0.015 | 1 | 0.015 | 56.32 | < 0.0001 | 10.71 |
| B-Volume of Eluent Solvent | 0.00067 | 1 | 0.00067 | 2.47 | 0.14 | 0.48 |
| C-Extraction Cycle | 0.00041 | 1 | 0.00041 | 1.49 | 0.24 | 0.29 |
| D-Elution Cycle | 0.0030 | 1 | 0.0030 | 11.00 | 0.0051 | 2.14 |
| AB | 0.0012 | 1 | 0.0012 | 4.48 | 0.053 | 0.86 |
| AC | 0.0001 | 1 | 0.0001 | 0.36 | 0.55 | 0.07 |
| AD | 0.0004 | 1 | 0.0004 | 1.46 | 0.25 | 0.29 |
| BC | 0.0012 | 1 | 0.0012 | 4.48 | 0.053 | 0.86 |
| BD | 0.0012 | 1 | 0.0012 | 4.48 | 0.053 | 0.86 |
| CD | 0 | 1 | 0 | 0 | 1.0000 | 0 |
| A^2 | 0.022 | 1 | 0.022 | 81.14 | < 0.0001 | 15/71 |
| B^2 | 0.066 | 1 | 0.066 | 241.87 | < 0.0001 | 47.14 |
| C^2 | 0.014 | 1 | 0.014 | 50.17 | < 0.0001 | 10 |
| D^2 | 0.020 | 1 | 0.020 | 74.36 | < 0.0001 | 14.29 |
| Residual | 0.0038 | 14 | 0.00027 |  |  |  |
| Lack of Fit | 0.0023 | 10 | 0.00023 | 0.63 | 0.74 |  |
| Pure Error | 0.0015 | 4 | 0.00037 |  |  |  |
| Cor Total | 0.11 | 28 |  |  |  |  |

% PC= Percent contribution, SS: sum of squares

The Model F-value of 166.66 (for CCD) and 28.37 (BBD) implies that the model is significant. A p-value lower than 0.001 was obtained, demonstrating again the high significance of the regression models. There is only a 0.01% chance that a "Model F-Value" could occur due to noise. Values of "Prob > F" less than 0.0500 indicate model terms are significant. Values greater than 0.1000 indicate the model terms are not significant. Data of ̏ prob > F ̋ less than 0.05 excessed which the models terms are important.

The predicated R^2^ of 0.9735 (for CCD) and 0.8591 (for BBD) were obtained. The value of adjusted R^2^ (0.9876 for CCD and 0.9319 for BBD) showed that only 1.24% (for CCD) and 6.81% (for BBD) of the total variations were not determined by CCD and BBD models.

Good relation between the real and predicted value indicated via the value of determination (R^2^= (0.9936 for CCD and 0.9659 for BBD). The lack-of-fit determined the failure of this model to represent values in regression. The non-significant value of lack-of-fit (>0.05) revealed that the model is statistically significant for this response. The lack-of-fit determined the failure of the model to represent values in the regression. The "Lack of Fit F-value" of 0.5596 (for CCD) and 0.6351 (for BBD) implies the Lack of Fit is not significant relative to the pure error.

For BBD: A, D, A^2^, B^2^, C^2^, D^2^ are significant model terms.

For CCD: A, B, D, AC, A^2^, B^2^, C^2^, D^2^ are significant model terms.

A great degree of precision and good deal of reliability of conducted runs were excessed applying a small data of the coefficient of variation (CV= 15.21 for BBD and 2.87 for CCD). The ANOVA of the regression model indicated that the quadratic model was significant, as was evident of Fisher ’s *F* test (*F*_model_= 166.66 (for CCD) and 28.37 (for BBD)) by a very low probability value (*p*) (*p*_model_= < 0.0001). Furthermore, F- value was calculated, that was obtained to be higher than the tabulated *F*-value (F*_a_*_, df, (n-df+1)_ = *F*_0.05, 14,17 (for CCD) and 16 (for BBD)_ = *F*_tab_ ≈ 2.3) at the 5 % level, explaining which the computed Fisher’s variance ratio at this level was large enough to justify a high degree of adequacy of quadratic model and also explained that the treatment combinations were highly important, as similarly proposed with Yetilmezsoy et al. [5]. Fig. SI is excessed two dimensional analytical response surfaces as the functions of two variables at the center level of other variables.

Eq (4) showed that Mallow’s C_p_ statistic can be applied to determine how many terms can be omitted of the response surface model. For a response surface model containing all terms, C_p_=p (where p is number of variable in the regression model). For a response surface model using omitted terms, C_p_ ≈ p indicates a good model by little bias and C_p_ ≤ p explains a very good prediction model. The goal is to delete terms of the response surface model until a minimum C_p_ ≈ p is achieved. If C_p_ > p this shows too many terms have been deleted or some remaining terms are not necessary.

C_p_= (SS_Residual_/MSS_Residual_) + 2p –n (eq. SI4)

Mallow’s C_p_ statistic (C_p_ ≈ 14.74 for CCD and 15.07 for BBD) indicated a third condition (C_p_ ≤ p and p = 15) explaining a very good prediction model for CCD. For BBD model, C_p_ > p that this shows too many terms have been deleted or some remaining terms are not necessary.

The result proved that the CCD method for optimization of the technique have suitable performance. So, the result of CCD method can be applied in further runs.

**References**

1. Hashemi SH, Ziyaadini M, Kaykhaii M, Jamali Keikha A, Naruie N. Separation and determination of ciprofloxacin in seawater, human blood plasma and tablet samples using molecularly imprinted polymer pipette-tip solid phase extraction and its optimization by response surface methodology. J Sep Sci. 2020;43:505–513.

2. Hashemi SH, Naruie N. Application of response surface methodology for pipette-tip micro solid-phase extraction of nicotine from cigarette, seawater and human plasma by a novel carbon nanotube/zinc oxide nanocomposite sorbent following its determination by spectrophotometry. J Anal Chem. 2021;76: 563–572.

3. Hashemi SH, Kaykhaii M, Jamali Keikha A, Naruie N. Application of molecularly imprinted polymer pipette tip micro‑solid phase extraction of nalidixic acid and acetaminophen from pills and seawater samples and their determination by spectrophotometry. Chem Papers. 2020;74:4009–4023.

4. Santelli RE, Bezerra MA, SantAna OD, Cassella RJ, Ferreira SLC. Multivariate technique for optimization of digestion procedure by focused microwave system for determination of Mn, Zn and Fe in food samples using FAAS. Talanta. 2006;68:1083–1088.

5. Yetilmezsoy K, Demirel S, Vanderbei RJ. Response surface modeling of Pb (II) removal from aqueous solution by Pistacia vera L.: Box-Behnken experimental design. J Hazard Mater. 2009;171:551–562.


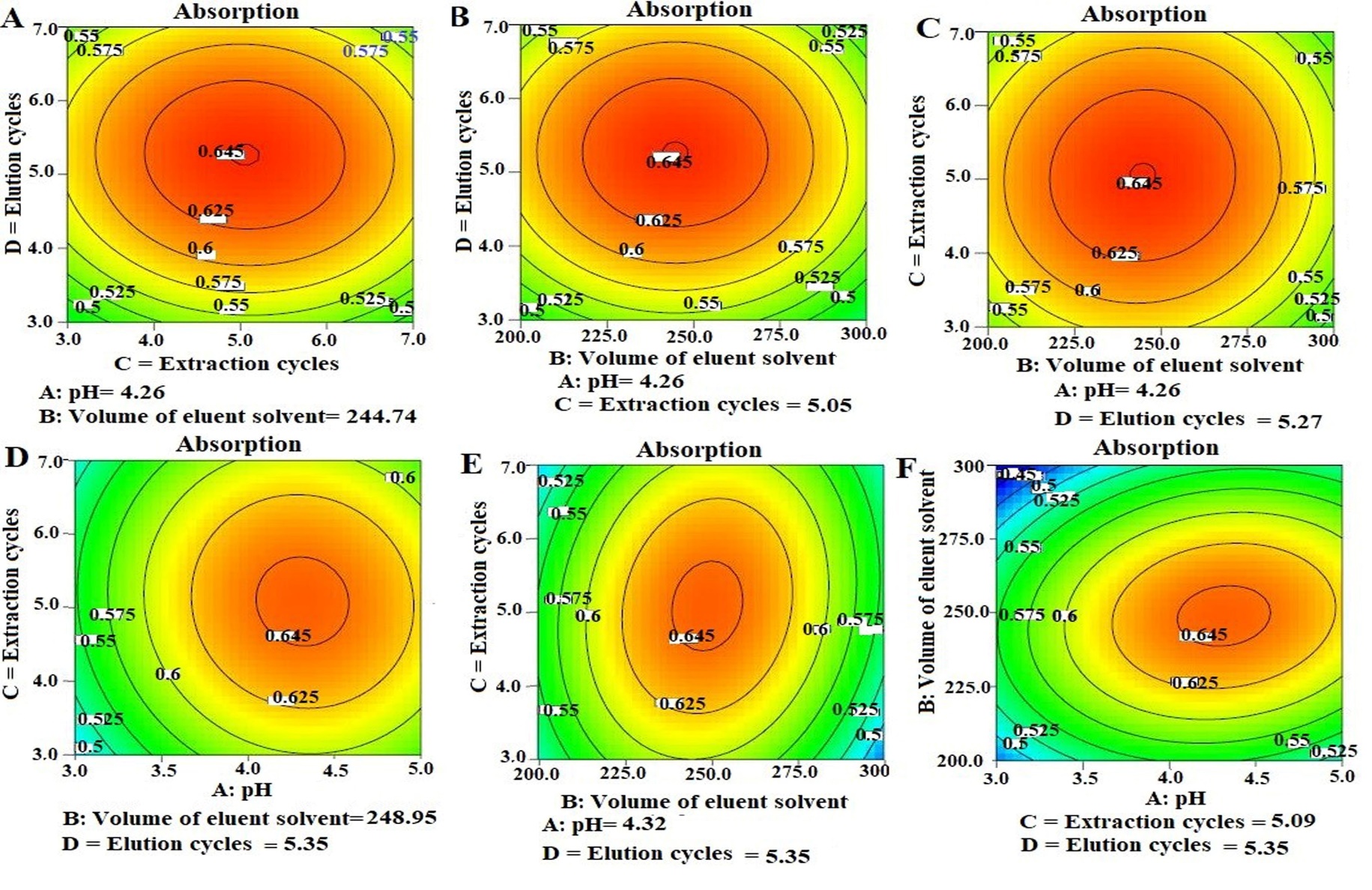


Fig. SI. Response surface -2D contours showing the effect of independent variable on absorbance. Two dimensional response surface as the functions of two variables center level of other variables are explained

1. ^1^Department of Marine Chemistry, Faculty of Marine Science, Chabahar Maritime University, Chabahar, Iran

   ^2^Department of Process Engineering and Chemical Technology, Faculty of Chemistry, Gdansk University of Technology, G. Narutowicza St. 11/12 80 – 233 Gdansk, Poland

   ^3^Department of Mechanical Engineering, Faculty of Marine Engineering, Chabahar Maritime University, Chabahar, Iran

   ^4^Mechanical Engineering Department, Faculty of Engineering, University of Sistan and Baluchestan, Zahedan, Iran

   *All correspondences should be sent to Massoud Kaykhaii, Tel: +48 731960312; E-mail: kaykhaii@gmail.com [↑](#footnote-ref-1)
